# Supplementary material for: Accurate recognition of colorectal cancer with semi-supervised deep learning on pathological images
Source: Nat Commun. 2021 Nov 2;12:6311. doi: 10.1038/s41467-021-26643-8 (PMC8563931; doi:10.1038/s41467-021-26643-8)
Supplement: Supplementary file 1 — Supplementary Information file [file 41467_2021_26643_MOESM1_ESM.pdf]

Supplementary information:

Accurate recognition of colorectal cancer with semi-supervised deep learning on pathological images

Gang Yu[1], Kai Sun[1], Chao Xu[2], Xing-Hua Shi[3], Chong Wu[4], Ting Xie[1], Run-Qi Meng[5], Xiang-He Meng[6], Kuan-Song Wang[7, #], Hong-Mei Xiao[6, #], Hong-Wen Deng[6,8, #]

A. Statistical analysis on patient-level prediction

The patient-level diagnosis was based on two strategies: cluster-based whole slide image (WSI) inference and positive sensitivity for patient inference. The main purpose of cluster-based WSI inference was to control the false positive rate (FPR) on each WSI, because the cancerous probability on each patch was not absolutely accurate, and multiple tests of many patches greatly increased false positives on one WSI.

Assuming the patch-level sensitivity and specificity were θ and γ, there were k consecutive positive patches on one WSI, i.e. the positive cluster size was k. At the same time, we also assumed that these patches were mutually independent. Theoretically, the probability of correctly identifying the WSI with one positive cluster was θ^k, and the probability of falsely identifying one WSI of non-cancer was $(1-\gamma)^k$. Assuming $k = 3$, γ=0.95, we had a patch-level FPR=0.05, while the FPR≈0.0001 on the WSI.

In practice, adjacent patches on a WSI were highly correlated. Consequently, the above theoretical derivation was not precise enough. However, the experiments proved that the false positive control of one WSI can achieve high predictive power with a cluster of four positive contiguous patches [1]. Therefore, we used the clustering of 4 patches as the condition for positive WSI inference. Finally, as long as the patient had a positive WSI, he or she was diagnosed with CRC (positive sensitivity).

Supplementary Table 1. Pathologist info full spelling here

Pathologist ID	Years in Clinic	Job Title
A	1	Resident physician
B	3	Resident physician
C	5	Physician-in-charge
D	7	Physician-in-charge
E	12	Physician-in-charge
F	18	Associate chief physician

Supplementary Table 2. List of area under the curve (AUC) of AI applied in CRC and other cancer types

Study	Patch-level test data		Independent patch-level test data		Slide-level test data		Independent slide-level test data		
	Number (#) of patches	AUC	# of patches	AUC	# of slides	AUC	# of datasets	# of slides	AUC
Colorectal cancer									
Haj-Hassan et al.[2]	NA	Unsegmented~0.7923<br><br>Segmented~0.9917	NA	NA	NA	NA	NA	NA	NA
Xu et al.[3]	717	0.969-0.980[a]	NA	NA	NA	NA	NA	NA	NA
Sari et al.[4]	1,592	0.994	NA	NA	NA	NA	NA	NA	NA
Kainz et al.[5]	60	0.983[a]	20[a]	0.950[a]	NA	NA	NA	NA	NA
Kather et al.[6]	100,000	0.987	7,180	0.943	NA	NA	NA	NA	NA
Ponzio et al.[7]	4500	0.9037-0.9682	NA	NA	NA	NA	NA	NA	NA
Shaw et al.[8], c	7,180	0.9377	NA	NA	NA	NA	NA	NA	NA
Model-10%-SSL	18,819	0.988-0.996	100,000	0.954-0.986	10,216[d]	0.984	11	1,967	0.946-0.990
Model-70%-SL	18,819	0.987-0.998	100,000	0.972-0.985	10,216[d]	0.990-0.992	11	1,967	0.957-0.990
Other cancers									
Coudray et al.[9] /lung cancer	NA	NA	NA	NA	244	0.990-0.993	3	340	LUAD~0.833-0.913<br><br>LUSC~0.861-0.941
Cruz-Roa et al.[10] / ductal carcinoma	50,963	0.842[b]	NA	NA	NA	NA	NA	NA	NA
Araujo et al.[11] /breast cancer	240	0.829[a]	192	0.693[a]	20	0.900[a]	1	16	0.750[a]
Motlagh et al.[12]/breast cancer	2,147	0.999	NA	NA	NA	NA	NA	NA	NA
Campanella et al.[13], e	NA	NA	NA	NA	12,132	0.986-0.991	1	12,727	0.986-0.991
Campanella et al.[13]	NA	NA	NA	NA	6,252	0.986-0.988	1	3,710	0.986-0.988
Campanella et al.[13]	NA	NA	NA	NA	8,670	0.965-0.966	1	1,224	0.965-0.966

Note: a: accuracy; b: balanced accuracy; c: semi-supervised learning, accuracy on training sets with 20% labeled data; d: XH-Dataset-PT and XH-Dataset-HAC.

e: AUC for prostate cancer, basal cell carcinoma and breast cancer metastases

Supplementary Table 3. Allocation (number) of Colorectal cancer (CRC) WSIs from 13 data centers

Dataset	PATT	PAT	PT	HAC
XH	842	0	10,216	213
NCT-UMM	0	86	0	0
TXH	0	0	135	135
PCH	0	0	96	96
HPH	0	0	99	99
FUS	0	0	198	198
GPH	0	0	185	185
SWH	0	0	199	199
AMU	0	0	205	205
SYU	0	0	97	97
ACL	0	0	207	207
CGH	0	0	100	0
TCGA-FFPE	0	0	446	0
Total	842	86	12,183	1,634

Supplementary Table 4. Hyper-parameters used in semi-supervised learning (SSL) and supervised learning (SL) of CRC

SSL	
Hyper-parameters	Value
Learning rate	0.0001
Optimizer	Adam
Epochs	500
Steps per epoch	100
Batch size	128
L2 decay	0.0001
Pre trained epochs	50
Early stopping	True
Patience	80
smoothing coefficient	0.95

SL	
Hyper-parameters	Value
Learning rate	0.001
decay rate	0.99
Optimizer	Adam
Epochs	500
Batch size	64
Steps per epoch	100
L2 decay	0.0001
Early stopping	True
Patience	50

Supplementary Table 5. Hyper-parameters used in SSL and SL of lung models

SSL	
Hyper-parameters	Value
Learning rate	0.0001
Optimizer	Adam
Epochs	500
Steps per epoch	100
Batch size	32
L2 decay	0.0001
Pre trained epochs	150
Early stopping	True
Patience	100
smoothing coefficient	0.9

SL	
Hyper-parameters	Value
Learning rate	0.001
decay rate	0.99
Optimizer	Adam
Epochs	500
Steps per epoch	100
Batch size	64
L2 decay	0.0001
Early stopping	True
Patience	50

Supplementary Table 6. Hyper-parameters used in SSL and SL of lymph node models

SSL	
Hyper-parameters	Value
Learning rate	0.0001
Optimizer	Adam
Epochs	500
Steps per epoch	200
Batch size	32
L2 decay	0.0001
Pre trained epochs	80
Early stopping	True
Patience	100
smoothing coefficient	0.9

SL	
Hyper-parameters	Value
Learning rate	0.001
decay rate	0.99
Optimizer	Adam
Epochs	500
Steps per epoch	300
Batch size	64
L2 decay	0.0001
Early stopping	True
Patience	50

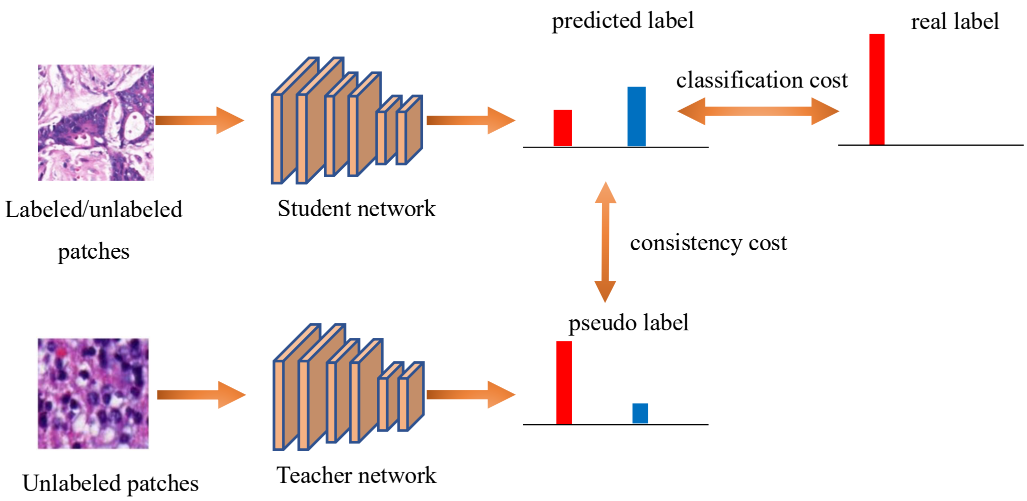

Supplementary Figure 1. The flow chart of mean teacher.

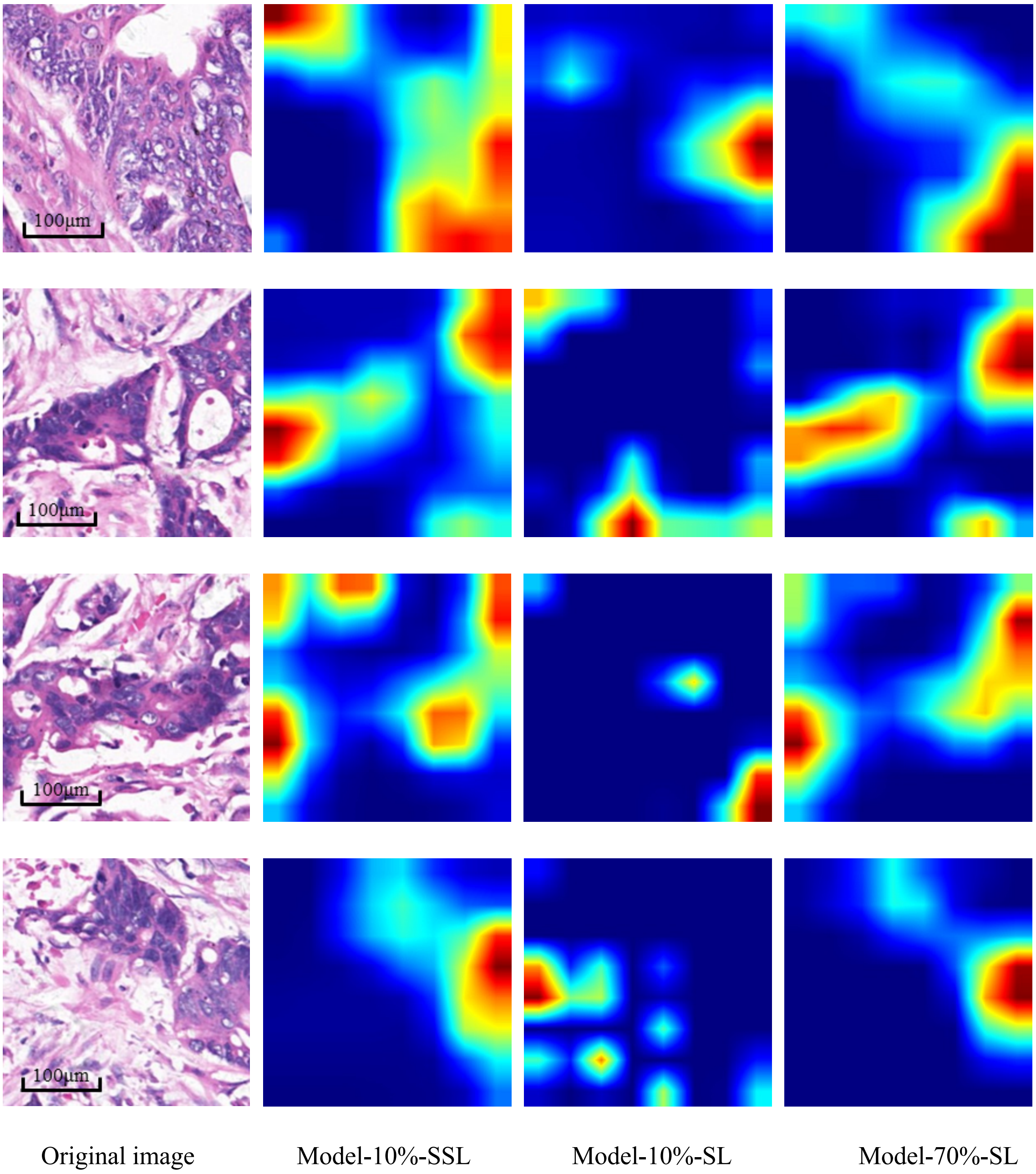

Supplementary Figure 2. Qualitative comparison of cancer locations in positive patches by visual inspection. The 50 positive patches (M=50) in the testing set were randomly selected. In the heatmaps, the pixels that had an important contribution to identifying cancers were shown with warm colors. The more important the pixel was for cancer recognition, the warmer the color (heatmap generated by [14]). Two senior and seasoned pathologists visually reviewed and accurately labeled the cancer location in the patches. If the pixels with warm color in generated heatmap included 70% of cancer locations labeled by pathologists, it was considered that the heatmap and the cancer locations were highly matched. This was because the heatmap roughly described the contribution of pixels to the recognition of cancer, rather than accurately segmenting the cancer.

All heatmaps (n=50, n/M=100%) generated by Model-10%-SSL or Model-70%-SL respectively are matched the location labeled by the pathologists. However, only a few heatmaps (n=17, n/M=34%) generated by Model-10%-SL are matched with the location labeled by pathologists, which shows that although the Model-10%-SL recognizes the patch as cancer, but it is not always based on the discovery of cancer locations. Four

mismatched samples are shown here, it can be seen that the activated regions of Model-10%-SSL and Model-70%-SL are very similar to each other. However, the heatmaps of Model-10%-SL are deviated from those of Model-10%-SSL and Model-70%-SL, both the size and location of regions with warm color in the patches.

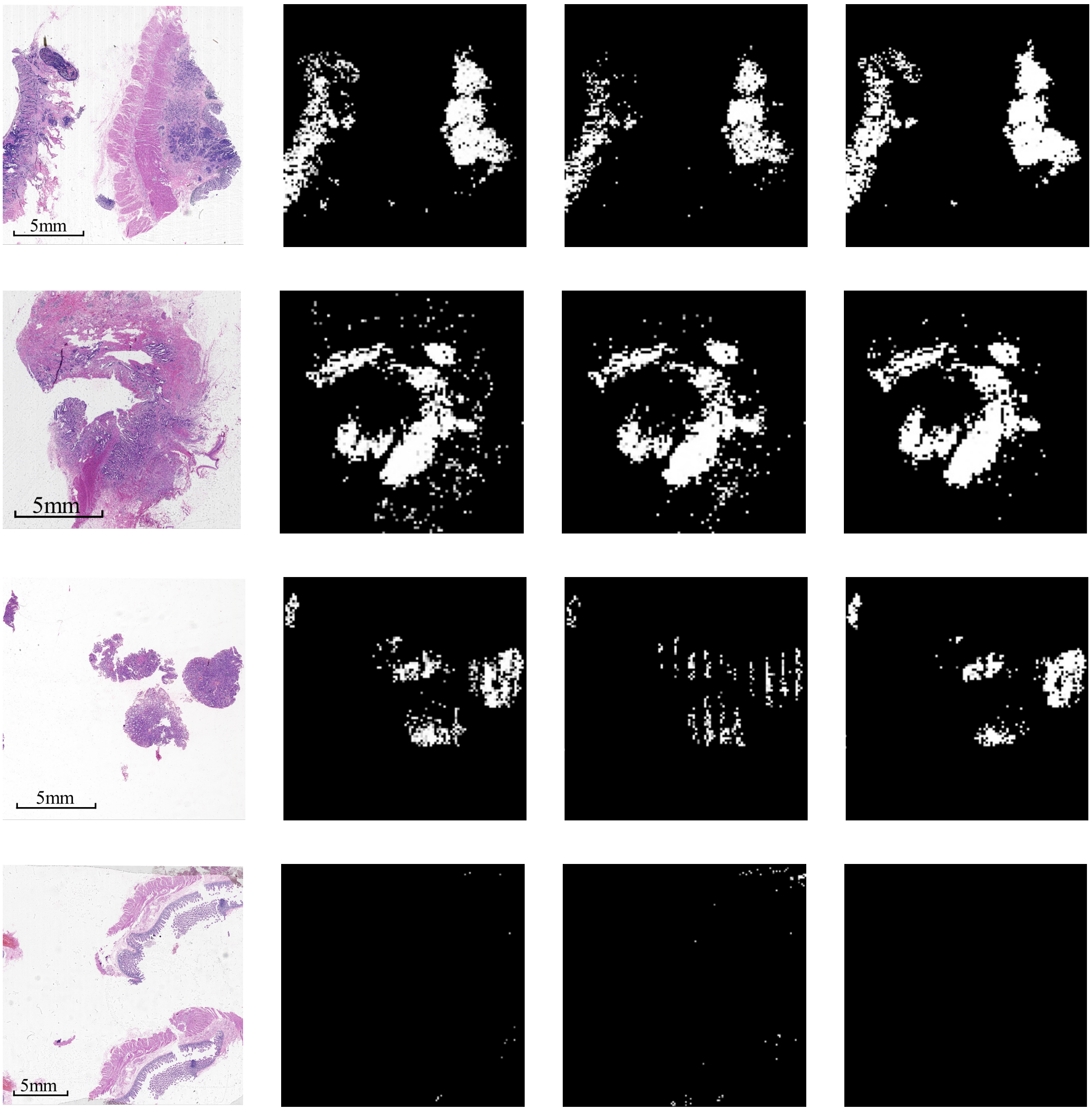

cropped from whole slide image   Model-10%-SSL   Model-10%-SL   Model-70%-SL

Supplementary Figure 3. Qualitative comparison of cancer locations in whole slide image (WSI) by visual inspection. The 50 (M=50) positive WSIs and 50 (M=50) negative WSIs were randomly selected from Dataset-PT. Two senior and seasoned pathologists visually reviewed and accurately labeled the cancer location in the 50 positive WSIs. For each positive WSI, the sensitivity (the number of positive patches correctly predicted by model divided by the number of positive patches labeled by pathologists) was calculated. If the sensitivity was greater than 90%, it was considered that the cancer location labeled by pathologists can be accurately found by the model. The heatmaps for highlighting predicted cancer regions (as patches) are shown in white (column 2, 3, 4).

In 50 positive WSIs, the sensitivity of all WSIs (n=50, n/M=100%) predicted by Model-10%-SSL or Model-70%-SL is greater than 90%, but only part of the sensitivity of WSIs (n=46, n/M=92%) predicted by Model-10%-SL is greater than 90%. In the heatmap of 50 negative WSIs predicted by Model-10%-SSL or Model-70%-SL, there are no cluster including four positive patches, which shows these negative WSIs (n=50,

n/M=100%) are correctly predicted. But in the heatmap of some negative WSIs (n=6, n/M=12%) predicted by Model-10%-SL, there are one or more clusters including four positive patches, which demonstrates these WSIs were incorrectly identified as positive.

Four samples are shown here, and the white regions in the heatmaps of three models are similar to each other and highly overlapped with the regions given by pathologists (row 1 and 2). However, some heatmaps of Model-10%-SL are deviated from those of Model-10%-SSL and Model-70%-SL (row 3 and 4). Moreover, the prediction of the negative WSI by Model-10%-SL (row 4) is cancerous, because there are some clusters including four cancerous patches in the heatmap.

Reference

[1] Wang, K.S., Yu, G., Xu, C. et al. Accurate diagnosis of colorectal cancer based on histopathology images using artificial intelligence. BMC Med. 19, 76 (2021).

[2] Haj-Hassan, H., Chaddad, A., Harkouss, Y. et al.. Classifications of multispectral colorectal cancer tissues using convolution neural network. J. Pathol. Inform. 8, 1 (2017).

[3] Xu, Y., Jia, Z., Wang, L.B. et al. Large scale tissue histopathology image classification, segmentation, and visualization via deep convolutional activation features. BMC Bioinformatics 18, 281 (2017).

[4] Sari, C.T, Gunduz-Demir, C. Unsupervised feature extraction via deep learning for histopathological classification of colon tissue images. IEEE transactions on medical imaging 38, 1139-1149 (2019).

[5] Kainz, P., Pfeiffer, M., Urschler, M. Segmentation and classification of colon glands with deep convolutional neural networks and total variation regularization. PeerJ 5, e3874 (2017).

[6] Kather, J.N., Krisam, J. Predicting survival from colorectal cancer histology slides using deep learning: a retrospective multicenter study. PLOS Med. 16, e1002730 (2019).

[7] Ponzio, F., Macii, E., Ficarra, E. et al. Colorectal cancer classification using deep convolutional networks-an experimental study. 5th International Conference on Bioimaging. (2018).

[8] Shaw, S., Pajak, M., Lisowska, A. et al. Teacher-student chain for efficient semi-supervised histology image classification. Preprint at arXiv:2003.08797v2 (2020).

[9] Coudray, N., Ocampo, P.S., Sakellaropoulos, T. et al. Classification and mutation prediction from non–small cell lung cancer histopathology images using deep learning. Nat. Med. 24, 1559–1567 (2018).

[10] Gurcan, M.N., Madabhushi, A., Cruz-Roa, A. et al. Automatic detection of invasive ductal carcinoma in whole slide images with convolutional neural networks. Proc. SPIE 9041, 904103 (2014).

[11] Araújo, T., Aresta, G., Castro, E. et al. Classification of breast cancer histology images using convolutional neural networks. PLOS ONE 12, e0177544 (2017).

[12] Jannesari, M., Habibzadeh, M., Aboulkheyr, H. et al. Breast cancer histopathological image classification: a deep learning approach. 2018 IEEE International Conference on Bioinformatics and Biomedicine, 2405-2412 (2018).

[13] Campanella, G., Hanna, M.G., Geneslaw, L. et al. Clinical-grade computational pathology using weakly supervised deep learning on whole slide images. Nat. Med. 25, 1301–1309 (2019).

[14] Selvaraju, R.R., Cogswell, M., Das, A. et al. Grad-CAM: visual explanations from deep networks via gradient-based localization. IEEE International Conference on Computer Vision. (2017).
