# Supplementary material for: Accurate recognition of colorectal cancer with semi-supervised deep learning on pathological images
Source: Nat Commun. 2021 Nov 2;12:6311. doi: 10.1038/s41467-021-26643-8 (PMC8563931; doi:10.1038/s41467-021-26643-8)
Supplement: Supplementary file 2 — Reporting Summary [file 41467_2021_26643_MOESM2_ESM.pdf]

nature portfolio

Corresponding author(s): Hong-Wen Deng

Last updated by author(s): 2021-10-9

Reporting Summary

Nature Portfolio wishes to improve the reproducibility of the work that we publish. This form provides structure for consistency and transparency in reporting. For further information on Nature Portfolio policies, see our Editorial Policies and the Editorial Policy Checklist.

Statistics

For all statistical analyses, confirm that the following items are present in the figure legend, table legend, main text, or Methods section.

n/a	Confirmed	
☐	☒	The exact sample size (*n*) for each experimental group/condition, given as a discrete number and unit of measurement
☐	☒	A statement on whether measurements were taken from distinct samples or whether the same sample was measured repeatedly
☐	☒	The statistical test(s) used AND whether they are one- or two-sided<br>*Only common tests should be described solely by name; describe more complex techniques in the Methods section.*
☒	☐	A description of all covariates tested
☒	☐	A description of any assumptions or corrections, such as tests of normality and adjustment for multiple comparisons
☐	☒	A full description of the statistical parameters including central tendency (e.g. means) or other basic estimates (e.g. regression coefficient) AND variation (e.g. standard deviation) or associated estimates of uncertainty (e.g. confidence intervals)
☐	☒	For null hypothesis testing, the test statistic (e.g. *F*, *t*, *r*) with confidence intervals, effect sizes, degrees of freedom and *P* value noted<br>*Give P values as exact values whenever suitable.*
☒	☐	For Bayesian analysis, information on the choice of priors and Markov chain Monte Carlo settings
☒	☐	For hierarchical and complex designs, identification of the appropriate level for tests and full reporting of outcomes
☒	☐	Estimates of effect sizes (e.g. Cohen's *d*, Pearson's *r*), indicating how they were calculated

Our web collection on statistics for biologists contains articles on many of the points above.

Software and code

Policy information about availability of computer code

Data collection	The details of data collection and annotation were described in the method section.
Data analysis	The algorithm pipeline were described in the method section. The code was available in the https://zenodo.org/record/5524324#.YU09Ny-KFLY, which is implemented by Python (3.8.10) and Tensorflow (2.3.1).

For manuscripts utilizing custom algorithms or software that are central to the research but not yet described in published literature, software must be made available to editors and reviewers. We strongly encourage code deposition in a community repository (e.g. GitHub). See the Nature Portfolio guidelines for submitting code & software for further information.

Data

Policy information about availability of data

All manuscripts must include a data availability statement. This statement should provide the following information, where applicable:

- Accession codes, unique identifiers, or web links for publicly available datasets
- A description of any restrictions on data availability
- For clinical datasets or third party data, please ensure that the statement adheres to our policy

Source Data is provided with this paper. The pathological images generated in this study have been deposited in the figshare database under accession code [https://doi.org/10.6084/m9.figshare.15072546.v1] [57], where the images of Dataset-PATT, Dataset-Lung and Dataset-Pcam can be used for patch-level retrain and retest. The independent patch-level testing set (Dataset-PAT) and 500 whole slide images in Dataset-PT have been provided with the source code for the patch-level and patient-level demo under accession code [https://zenodo.org/record/5524324#.YU09Ny-KFLY]. The remaining WSIs in Dataset-PT and Dataset-HAC can be obtained by contacting the corresponding author by the Email [Kuan-Song Wang <375527162@qq.com>]. All data access in this study can only be requested by the researchers and for scientific research purposes. The data access requests will be processed in ten business days.

Field-specific reporting

Please select the one below that is the best fit for your research. If you are not sure, read the appropriate sections before making your selection.

☒ Life sciences ☐ Behavioural & social sciences ☐ Ecological, evolutionary & environmental sciences

For a reference copy of the document with all sections, see nature.com/documents/nr-reporting-summary-flat.pdf

Life sciences study design

All studies must disclose on these points even when the disclosure is negative.

Sample size	13,111 colorectal cancer(CRC) whole slide images from 8,803 subjects; 15,000 lung and 294,912 lymph node images. For every model on CRC , 8 experiments (sample/patch number per experiment=162,919) at patch-level; three model experiments on 12 center data (WSI number per experiment=12,183) at patient-level; For every model on lung, 8 experiments (sample/patch number per experiment=15,000); For every model on lymph node, 8 experiments (sample/patch number per experiment=294,912). The sample size are massive, so the area under the curve or accuracy of each experiment are confident. The Wilcoxon signed rank test (sample size or experiment number/group=8, 12, 16) is then performed on the significant difference of area under the curve or accuracy at patch-level and patient-level.
Data exclusions	For data from hospitals, the data were reviewed by pathologists. If a consensus was not reached, the data were excluded. For data from public database, no data were excluded. We described the data collection and exclusions in details in the method.
Replication	We have proved that the results can be replicated in the three diseases using external datasets, both at the patch level and the patient level pathological images.
Randomization	In the data collection, the technicians randomly selected slides from archive library. The number of selected patients collected on the same day was limited to less than 50 to make sure the selected images for this study were not unduly influenced by samples collected on any one single day. so the samples are random.
Blinding	Data collectors and pathologists for annotations do not participate in follow-up studies. The computer randomly allocates the datasets, and the experts participating in the evaluation did not participate in the methodological research. Therefore, investigators are blind to the data.

Reporting for specific materials, systems and methods

We require information from authors about some types of materials, experimental systems and methods used in many studies. Here, indicate whether each material, system or method listed is relevant to your study. If you are not sure if a list item applies to your research, read the appropriate section before selecting a response.

Materials & experimental systems

n/a	Involved in the study
☒	☐ Antibodies
☒	☐ Eukaryotic cell lines
☒	☐ Palaeontology and archaeology
☒	☐ Animals and other organisms
☒	☐ Human research participants
☒	☐ Clinical data
☒	☐ Dual use research of concern

Methods

n/a	Involved in the study
☒	☐ ChIP-seq
☒	☐ Flow cytometry
☒	☐ MRI-based neuroimaging
